# Retinal Microvascular Alterations in Diabetic Patients Assessed with Novel Imaging Techniques

**DOI:** 10.3390/life16071095

**Published:** 2026-06-30

**Authors:** Martyna Liśkiewicz-Jankowska, Edyta Dąbrowska, Jacek Wolf, Krzysztof Narkiewicz

**Affiliations:** 1 Department of Hypertension and Diabetology, Medical University of Gdansk, Dębinki 7, 80-211 Gdansk, Poland; jacek.wolf@gumed.edu.pl (J.W.); krzysztof.narkiewicz@gumed.edu.pl (K.N.); 2 Translational Medicine Centre, Medical University of Gdansk, Dębinki 7, 80-211 Gdansk, Poland; edabrowska@gumed.edu.pl

**Keywords:** diabetes mellitus, retinal microcirculation, microvascular dysfunction, scanning laser Doppler flowmetry, adaptive optics, optical coherence tomography angiography, wall-to-lumen ratio, cardiometabolic risk, laser speckle flowgraphy

## Abstract

The global burden of diabetes mellitus and its chronic complications—including premature atherosclerotic cardiovascular disease and microvascular impairments such as nephropathy, retinopathy, and neuropathy—highlights the need for improved strategies for the early identification of individuals at risk of carbohydrate metabolism disorders. Growing evidence suggests that alterations in the microcirculatory bed occur concomitantly with, or may even precede, the development of cardiovascular disease. The eye, owing to its transparent anatomical structures, provides a unique opportunity for the in vivo assessment of microvascular changes and offers valuable insights into other vascular territories, serving as a potential “window” into cardiometabolic disorders. Recent advances in microcirculation imaging have enabled detailed, non-invasive evaluation of the retinal microvasculature. Techniques such as scanning laser Doppler flowmetry (SLDF), adaptive optics (AO), optical coherence tomography angiography (OCTA), and laser speckle flowgraphy (LSFG) allow for quantitative assessment of the retinal microvascular bed, demonstrating partial correlation with invasive measures of vascular function and sensitivity to therapeutic interventions. The integration of these imaging modalities into clinical and research settings may facilitate the early detection of microvascular dysfunction, improve risk stratification, and support the monitoring of disease progression and treatment efficacy in patients with carbohydrate metabolism disorders. Therefore, this review aims to summarize the current evidence on retinal microvascular alterations in carbohydrate metabolism disorders assessed using advanced imaging techniques, focusing primarily on early, subclinical retinal changes that precede the onset of diabetic retinopathy.

## 1. Introduction

It is estimated that approximately 425 million people worldwide are affected by diabetes mellitus, encompassing type 1 (T1D) and type 2 (T2D). If the current trend continues, the disease prevalence is predicted to increase to approximately 693 million people (10% of the global adult population) by 2045 [1]. It is of crucial importance, as diabetes causes numerous chronic complications affecting large vessels, leading to premature atherosclerotic cardiovascular diseases [2], which are the primary cause of death in this group of patients. In addition, diabetes contributes to microvascular complications such as nephropathy, retinopathy, and neuropathy. Consequently, expenditures on diabetes-related health care are constantly rising, reflecting the growing social, financial, and health burdens associated with the disease [3].

The identification of novel biomarkers could enable improved risk stratification and therapeutic intervention at the subclinical stage of the disease. It has been demonstrated that microcirculatory impairments occur simultaneously with [4,5] or may even precede cardiovascular diseases [6].

The eye, composed of transparent anatomical structures, represents a unique and readily accessible place for microcirculation evaluation. Emerging, promising non-invasive techniques for retinal microvascular assessment can provide valuable insights into changes occurring in other microvascular beds and potentially serve as a window into cardiovascular and metabolic diseases, including diabetes [7]. This review focuses primarily on subclinical microvascular alterations that precede or accompany the onset of diabetic retinopathy, rather than on established retinopathy itself.

## 2. Microcirculatory Bed—Pathophysiological Meaning

Microcirculation is the terminal part of the circulatory system, forming a network that includes vessels less than 150 µm in diameter, which can be divided into five main vascular areas: small arteries (internal diameter from 100 to 300 µm), arterioles (7–100 µm), capillaries (around 7 µm), as well as small veins and venules—each of them with unique anatomical, physiological and regulatory properties [8]. According to Poiseuille’s law, vascular resistance is inversely proportional to the fourth power of vessel radius; thus, even slight functional or structural alterations affecting the arteriolar lumen may result in significant changes in arterial resistance [9]. Therefore, in view of the dimensions of microcirculatory bed vessels and their substantial total area—estimated at 500–700 m^2^ [10]—microcirculation acts as a pivotal modulator of the total peripheral resistance [11] and plays an essential role in the interplay between the remaining components of the circulatory system, such as macrocirculation and the heart [12].

Previous studies have revealed associations between retinal, cardiac [13], and cerebral microcirculation [14]; therefore, retinal vessel alterations might be considered as a surrogate measure of other vascular districts long before clinical manifestation of cardiometabolic diseases [8].

Several interrelated pathophysiological processes both contribute to and arise from endothelial dysfunction. Circulating and cellular mediators of endothelial dysfunction—such as elevated vascular cell adhesion molecules (VCAM-1, ICAM-1), increased NADPH oxidase activity, and reactive oxygen species (ROS) formation—promote inflammation and oxidative stress. These processes reduce nitric oxide (NO) bioavailability, reducing vasodilatory potential and enhancing prothrombotic responses. These alterations, known as small vessel disease, are associated with structural remodeling and microvascular rarefaction, causing reduced blood supply and end-organ damage [15].

Considering arterial remodelling and its influence on the lumen diameter, it can be either inward or outward. Changes observed in the vascular wall include hypertrophic (thickening of the vascular wall), eutrophic (rearrangement of smooth muscle cells, maintaining the same wall cross-sectional area), or hypotrophic remodelling (thinning of the vascular wall) [16].

While outward hypertrophic remodelling is mainly seen in large central elastic arteries [17], inward remodelling is commonly observed in small resistance arteries and is caused by a thickened arterial wall together with a reduced lumen diameter, resulting in an elevated wall-to-lumen ratio (WLR) [18]. Moreover, depending on the disease entity, inward remodelling divides into eutrophic or hypertrophic [19]. Additionally, rarefaction manifests as structural or functional and concerns the most distal part of the microcirculation—arterioles and capillaries [18].

Diabetes has been shown to induce heterogeneous alterations in microvascular vessel dimensions [12]. Moreover, it has been proven that retinal arteriolar narrowing has a prognostic value for the development of hypertension [20]. Furthermore, increasing evidence suggests that retinal vessel phenotypes are associated with the development of neurodegenerative and cerebrovascular diseases [14]. The retinal microvascular network has also been described as a window to the heart [21]. Hence, retinal microvascular biomarkers can potentially serve as a diagnostic tool for systemic cardiovascular risk prediction.

## 3. Retinal Microcirculation in Diabetes

For the past few decades, the link between microvasculature and carbohydrate metabolism disorders has been widely investigated. Emerging evidence from large cohort studies demonstrates complex, heterogeneous vessel dimension alterations in diabetes—arteriolar, as well as venular remodelling [12].

Diabetes-related changes in retinal vessel diameter result from the pathophysiological effects of hyperglycaemia and inflammation on the retinal microvasculature. Increased blood glucose levels in diabetes are associated with pericyte loss mediated by glycation, inflammatory processes, and the induction of oxidative stress [22,23].

Notably, the retinal endothelium is particularly susceptible to oxidative stress, as it generates more deleterious superoxide and exhibits reduced activity of the protective enzyme superoxide dismutase [24]. In addition, acute elevations in blood glucose levels have been shown to impair myogenic constriction of retinal arterioles [25]. The combined effects of smooth muscle cell loss and impaired myogenic constriction under hyperglycaemic conditions may underlie the association between retinal arteriolar widening and diabetes. Consistent with these mechanisms, several cross-sectional cohort studies have reported wider retinal arterioles in individuals with diabetes, including the Singapore Malay Eye Study (N = 3280) [26] and the Multi-Ethnic Study of Atherosclerosis (N = 5976) [27]. Furthermore, in the Maastricht Study (N = 2876), retinal arteriolar widening was also associated with higher HbA1c levels [28].

Heterogeneous alterations of retinal vessel dimensions in diabetes encompass not only arteriolar widening but also arteriolar narrowing. Although these seemingly contradictory findings may appear confusing, they likely reflect distinct pathophysiological mechanisms that evolve over the natural course of diabetes [8].

Arteriolar dilation and retinal hyperperfusion are considered early and relatively short-term vascular manifestations, predominantly reported in cross-sectional cohort analyses. In contrast, retinal arteriolar narrowing appears to represent a more advanced stage of retinal microvascular impairment, as demonstrated in prospective follow-up cohort studies [8]. This pattern was clearly demonstrated in the AusDiab Study (N = 803), where baseline cross-sectional analyses showed an association between wider retinal arterioles and prevalent diabetes [29], whereas narrower retinal arteriolar calibre independently predicted incident diabetes over a 5-year follow-up period [30]. Similarly, in the Atherosclerosis Risk in Communities Study (N = 7993), the incidence of diabetes was associated with narrower arterioles, expressed by a lower arteriolar-to-venular ratio (AVR) at baseline [31].

In addition, alterations in retinal venular calibre have been observed. With increasing inflammatory burden, disease duration, and age, diabetes is frequently associated with retinal venular widening. This association was demonstrated in the prospective Rotterdam Study (N = 2309), in which wider retinal venules were independently associated with an increased risk of impaired fasting glucose and incident diabetes [32].

Beyond changes in outer vessel diameter, advanced imaging techniques have enabled the assessment of vascular wall structural alterations in diabetes, revealing evidence of hypertrophic remodelling [12]. Repeated acute hyperglycaemia, leading to impaired myogenic response [25], results in increased wall stress at a given intraluminal pressure, which may promote vascular hypertrophy [33]. Moreover, functional vascular dysfunction is already evident in prediabetes, as demonstrated by reduced retinal flicker light–induced vasodilation [34].

Another microcirculatory alteration observed in the course of diabetes is microvascular rarefaction [35], which represents a reduction in functional capillary density leading to impaired tissue perfusion and increased microvascular resistance. Taken together, these observations support the concept of dynamic, stage-dependent remodelling of the retinal microvasculature in diabetes.

Importantly, arterial hypertension (AH) frequently coexists with diabetes and exerts its own pronounced effects on the retinal microvasculature, so that retinal arteriolar remodelling in diabetic patients cannot be attributed to hyperglycaemia alone. Both conditions independently increase the wall-to-lumen ratio of retinal arterioles [12,36] but they appear to predominate at different stages: recent adaptive optics data suggests that AH seems to be the principal driver of arteriolar wall thickening in the preclinical phase—before overt diabetic retinopathy—whereas diabetes itself becomes the predominant determinant once clinical retinopathy develops [37]. Furthermore, AH impairs retinal vascular autoregulation, and this dysfunction appears to be exacerbated in patients with type 2 diabetes [38]. Blood-pressure status should therefore be regarded as an essential covariate when interpreting retinal microvascular phenotypes in diabetes, with the two conditions acting synergistically rather than independently [35].

In light of accumulating evidence that microvascular dysfunction may precede and contribute to the development of T2D [39], recent advances in retinal microvascular imaging offer promising opportunities to enhance diagnostic precision and improve the management of carbohydrate metabolism disorders.

## 4. Novel Imaging Techniques

For decades, micromyography has been the gold standard for evaluating microvascular structural and functional alterations by dissection of subcutaneous small vessels from tissue biopsies [18]. Micromyography’s disadvantages—invasiveness and ex vivo assessment—drove the development of non-invasive and easy-to-perform techniques to assess the microvascular phenotype in vivo. Retinal microvasculature assessment, owing to the transparency of anatomical structures of the eyeball, has met those criteria and enables real-time retinal examination [40].

**Figure 1:**
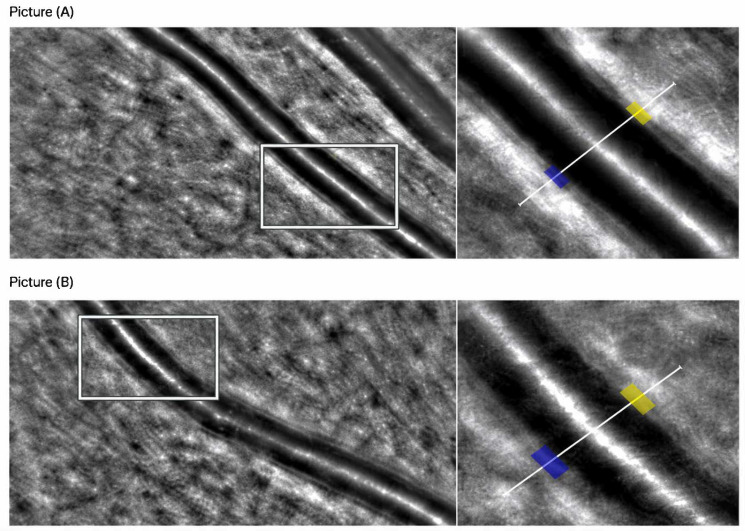
Comparison of retinal arteriolar WLR measured in 2 subjects by adaptive optics (rtx1 camera, Imagine Eyes, Orsay, France). (**A**) Left eye, retina temporal superior from optic nerve of 27-year-old woman without chronic diseases- WLR 0.21. (**B**) Right eye, retina temporal superior from optic nerve of 64-year-old male patient with a long-standing history of hypertension, T2DM, and dyslipidemia, receiving multidrug antihypertensive therapy WLR 0.39. Examination, all measurements and analysis were done in the same conditions (5 min after rest in sitting position in dark room without mydriasis). Source: authors’ own archive.

In this article, SLDF, AO, OCTA, and laser speckle flowgraphy (LSFG) are discussed with respect to their use principles, applications, and potential relevance in the assessment of cardiometabolic and diabetes-related microvascular alterations. A head-to-head comparison of their technical and clinical features is provided in Table 1, and a representative side-by-side AO comparison of a healthy versus diseased retinal arteriole is shown in Figure 1. A summary of the key clinical studies discussed in this review is provided in Table 2.

### 4.1. Scanning Laser Doppler Flowmetry (SLDF)

SLDF was the first method to provide non-invasive, in vivo quantification of the retinal arteriolar wall-to-lumen ratio and generated much of the foundational evidence linking retinal arteriolar remodelling to cardiometabolic risk. This technique enables the assessment of structural and functional parameters of retinal microcirculation. Confocal microscopy is used to measure outer vessel diameter, whereas the laser Doppler effect is used to analyse lumen diameter and retinal capillary blood flow (RCF). Wall thickness is calculated by subtracting the lumen dimension from the outer diameter of the retinal vessel. The examination is performed in a dark room, after 15 min of rest, without the need for any pharmacological pupil dilatation. The measurement is taken in the juxtapapillary area of the right eye, 2–3 mm temporally superior to the optic nerve [46]. Additionally, using flicker-light with a frequency of 8 Hz, dynamic alterations in retinal blood flow velocity may be analysed [47]. Population-based reference data indicate that retinal arteriolar wall-to-lumen ratio (WLR) assessed by scanning laser Doppler flowmetry (SLDF) is approximately 0.31 ± 0.07 in healthy individuals. Higher WLR values were observed with increasing age [48].

Multiple, clinically relevant insights have been obtained using SLDF measurements. An increased WLR has been observed in hypertensive patients with a history of cerebrovascular event [5] or with cardiac damage [49], as well as in patients with hypertension or diabetes [50], and in those with heart failure or elevated urinary albumin excretion [51].

Regarding RCF, it is reduced in T1D (gradually decreasing with disease duration) [41], whereas in T2D, preclinical retinopathy is associated with increased RCF, potentially reflecting a higher risk of progression [42].

It has been demonstrated that SLDF provides information on microvascular morphology comparable to that obtained with invasive measurements [52]. Unfortunately, the Heidelberg Retina Flowmeter is no longer commercially available, which has limited its promising clinical development [53].

### 4.2. Adaptive Optics Ophthalmoscopy

The recently developed non-invasive in vivo approach—the rtx1 camera (Imagine Eyes, Orsay, France)—utilises an adaptive optics technique, originally designed by Horace Babcock in 1953 for astronomical telescopes to eliminate the aberrations generated by the atmosphere [54].

In the eye, corresponding wavefront aberrations are generated by the various refractive surfaces in the anterior optics of the eyeball. In adaptive optics, these defects are corrected in real time by a deformable mirror, enabling previously inaccessible, precise imaging with a resolution of about 1 µm [55]. The obtained image visualises the photoreceptors, capillaries, arterioles, and venules [56,57]. The application of adaptive optics in the clinical evaluation of retinal microcirculation is presented in Figure 1. It should be emphasised that adaptive optics is not an imaging modality in itself but a wavefront-correction technology combined with an underlying imaging system; besides the flood-illumination fundus camera used here (rtx1), it can be coupled with scanning laser ophthalmoscopy (AO-SLO) and optical coherence tomography (AO-OCT) [55,56].

The coordinates of the region of interest are automatically retained, allowing for comparison of measurements at subsequent visits and analysis of disease development or response to treatment.

Compared to SLDF, AO detects retinal arteriole remodelling with a lower intra-observer variability [58] and provides very high-quality images [59]. Recently, the comparison of the media-to-lumen ratio (MLR) of subcutaneous small resistance arteries measured by wire micromyography with WLR evaluated by either AO or SLDF has shown a clear advantage of AO over SLDF [58]. This is especially important, since these two techniques were reported as a promising approach in the 2023 ESH Guidelines for the management of AH-mediated organ damage [60].

Retinal imaging assessed by AO has provided clinically relevant results. Most studies employing the rtx1 camera have reported a significantly higher wall-to-lumen ratio in individuals with diabetes without retinopathy compared with healthy controls [36,43]. Furthermore, abnormalities detected with adaptive optics indicate early arteriolar dysfunction that may precede progression from impaired glucose tolerance to overt diabetes, underscoring the potential of this emerging technique to non-invasively identify subclinical vascular changes beyond the capabilities of routine clinical examination [44]. In adaptive optics studies, retinal arteriolar WLR values in healthy controls have generally ranged from 0.23 to 0.29 [61]; however, standardized reference ranges remain unavailable. Recent data obtained using flood-illumination AO imaging with the rtx1 camera by Kortuem et al. reported lower values (0.164 ± 0.019 for central arterioles and 0.185 ± 0.026 for peripheral branches), emphasizing the need for method-specific normative values [62].

Diabetic retinopathy (DR) has also been extensively investigated using AO. This common (approximately one-third of patients) microvascular complication of diabetes, if untreated, leads to vision impairment, including blindness—the main cause of vision loss in working-age people [63]—and remains a predictor of life-threatening macrovascular complications, including stroke, coronary heart disease, heart failure, and nephropathy [64]. This association is more consistently seen in patients with T2D as compared to T1D, reflecting older age and possibly the higher prevalence of cardiovascular risk factors in T2D [65]. In clinical terms, adaptive optics (AO) imaging may provide additional insights into diabetic retinopathy by enabling visualization of retinal microvascular and neural alterations at near-cellular resolution that are beyond the capabilities of conventional fundus examination. AO allows detailed assessment of microaneurysms, focal arteriolar wall thickening, and cone photoreceptor abnormalities, potentially facilitating earlier detection of disease-related changes, objective evaluation of severity, and longitudinal monitoring of progression and treatment effects [36,43,66].

### 4.3. Optical Coherence Tomography Angiography (OCTA)

Optical coherence tomography angiography is a recently developed imaging route that enables a 3-dimensional representation of retinal flow, using signal changes caused by moving blood cells—such as erythrocytes—in series of repeated B-scans [18]. This approach does not require dye injections, permitting depth-resolved visualisation of retinal and choroidal vasculature, which is the biggest advantage of this method [56]. Moreover, OCTA has been compared with conventional fluorescein angiography, which is considered the criterion standard for the assessment of retinal vasculature. The imaging findings of the retinal vascular layers demonstrated a clear advantage of OCTA over fluorescein angiography [67]. The technical principles, advantages, limitations, and device-related specifications of OCTA have been comprehensively reviewed by Koutsiaris et al. [68].

In diabetes, OCTA has provided novel pathophysiological insights, with detection of vascular alterations in subjects with prediabetes and diabetes even before the onset of symptomatic retinopathy. Clinically, OCTA may enhance the assessment of retinal microvascular alterations by revealing early subclinical changes in patients with diabetes and prediabetes, including those without clinically apparent retinopathy. These microvascular abnormalities may coexist with neuroretinal dysfunction and precede overt retinal changes. OCTA applications include the assessment of capillary non-perfusion, detection of retinal neovascularization, and risk stratification for retinopathy progression or development of diabetic macular oedema [45]. Normative OCTA databases have established reference values for macular vessel density and foveal avascular zone parameters. Age-related declines in retinal capillary density emphasize the need for age-matched reference values when interpreting OCTA findings in diabetes [69,70]. However, OCTA has several major limitations, including motion and projection artifacts, segmentation errors, and signal attenuation [71].

### 4.4. Laser Speckle Flowgraphy (LSFG)

Laser speckle flowgraphy (LSFG) is a non-invasive technique that assesses ocular blood flow dynamics based on laser speckle patterns generated by light scattered from moving erythrocytes. Its principal parameter, the mean blur rate (MBR), provides a relative and highly reproducible index of blood flow velocity in the optic nerve head, retina, and choroid [72]. Normative MBR values have been established in healthy populations and demonstrate age-related variability [73]. In diabetes, LSFG studies have reported altered retinal and optic nerve head perfusion, with changes associated with diabetic retinopathy severity and treatment-related haemodynamic modifications [74]. Limitations include the expression of MBR in arbitrary units, limiting direct comparisons between devices, and sensitivity to ocular and biometric factors. LSFG therefore may complement the structural information provided by SLDF and AO with a rapid, functional assessment of ocular perfusion.

### 4.5. Artificial Intelligence in Retinal Microvascular Imaging

The clinical utility of retinal microvascular imaging increasingly depends on reproducible, observer-independent quantitative analysis, an area in which artificial intelligence (AI) is playing a growing role. Deep-learning algorithms enable automated segmentation of retinal vascular structures and quantification of microvascular biomarkers, including vessel density and foveal avascular zone (FAZ) metrics. Recent transformer-based models incorporating knowledge distillation have demonstrated robust automated FAZ segmentation on OCTA images across multiple retinal conditions, including diabetic retinopathy [75]. Beyond image quantification, AI applied to retinal imaging has shown the ability to predict systemic cardiovascular risk factors and major adverse cardiovascular events directly from fundus photographs, indicating that retinal images may contain clinically relevant information on systemic health that is not readily discernible by conventional assessment. These findings support the emerging concept of retinal imaging as a source of novel biomarkers for cardiometabolic risk stratification and precision medicine [76].

## 5. Conclusions

The growing global burden of diabetes mellitus and its chronic complications underscores the urgent need for improved strategies aimed at the early identification of individuals at risk of carbohydrate metabolism disorders. Early detection of microvascular impairment may provide valuable opportunities for timely intervention, thereby mitigating the progression of systemic complications associated with diabetes. Recent advances in non-invasive retinal imaging modalities have the potential to complement current diagnostic approaches, refine risk stratification, and contribute to a more individualised management of patients with diabetes. Future research is warranted to validate these techniques and adapt them for clinical use. Such efforts may ultimately improve therapeutic outcomes and inform precision-based prevention and treatment strategies in diabetes care.

## Figures and Tables

**Table 1:** Comparison of four non-invasive retinal microvascular imaging techniques applied in cardiometabolic and diabetic microvascular research. Ø, diameter; OCTA, optical coherence tomography angiography; RCF, retinal capillary blood flow; SLDF, scanning laser Doppler flowmetry; WLR, wall-to-lumen ratio. LSFG, laser speckle flowgraphy; MBR, mean blur rate.

Feature	SLDF	Adaptive Optics (rtx1)	OCTA	LSFG
**Imaging principle**	Confocal scanning + laser Doppler shift from moving erythrocytes	Real-time wavefront correction by deformable mirror	Repeated B-scans; motion contrast between scans	Laser speckle contrast from moving erythrocytes (full-field, no scanning)
**Spatial resolution**	~10 µm (vessel level)	~1 µm (single-cell, photoreceptors)	~5–15 µm in-plane; depth-resolved layers	Flow maps over regions (ONH, vessels, choroid); no single-vessel wall resolution
**Parameters measured**	Outer Ø, lumen Ø, wall thickness, WLR, retinal capillary blood flow (RCF)	Outer Ø, lumen Ø, WLR; cone density and regularity	Vessel density, foveal avascular zone, layer-specific perfusion	Mean blur rate (MBR)—relative blood-flow velocity
**Region of interest**	Juxtapapillary area (2–3 mm temporal–superior to optic nerve)	Macular and peripapillary arterioles ~80–120 µm	Superficial, deep, and choroidal vascular plexuses	Optic nerve head, retinal vessels, choroid
**Pupil dilation**	Not required	Not required	Usually not required	Not required
**Dye injection**	No	No	No	No
**Main limitations**	Operator-dependent; Heidelberg Retina Flowmeter no longer commercially available	Lower throughput; learning curve	Motion/projection artifacts; segmentation errors; signal attenuation in deeper layers	Relative (arbitrary) units; sensitive to ocular pressure and axial length; limited wall-structure data
**Commercial availability (2026)**	Discontinued	Yes (Imagine Eyes rtx1)	Yes (multiple vendors)	Yes (Nidek LSFG-NAVI)

**Table 2:** Summary of key clinical studies on retinal microvascular alterations in carbohydrate metabolic disorders discussed in this review. AO, adaptive optics; AVR, arteriolar-to-venular ratio; DR, diabetic retinopathy; FAZ, foveal avascular zone; OCTA, optical coherence tomography angiography; RCF, retinal capillary blood flow; SLDF, scanning laser Doppler flowmetry; T1D/T2D, type 1/type 2 diabetes; WLR, wall-to-lumen ratio.

Study	Cohort/Number	Technique/Parameter	Key Observation
Singapore Malay Eye Study; Islam 2009 [26]	3004 Malays (40–80 y)	Fundus photography; retinal vascular caliber	Diabetes and DR associated with retinal vascular caliber abnormalities, particularly venular widening.
MESA; Nguyen 2008 [27]	5976 adults; 892 with diabetes	Fundus photography; CRAE, CRVE	Diabetes associated with wider arterioles and venules; venular widening correlated with DR.
Maastricht Study; Li 2020 [28]	3468 participants	Fundus photography; CRAE	T2D and higher HbA1c independently associated with wider retinal arterioles.
AusDiab; Tikellis 2007 [29]	2476 participants	Fundus photography; CRAE, CRVE	Diabetes associated with wider retinal vessels; venular changes correlated with retinopathy.
AusDiab 5-y follow-up; Nguyen 2008 [30]	803 non-diabetic participants	Fundus photography; CRAE	Retinal arteriolar narrowing predicted incident diabetes.
ARIC; Wong 2002 [31]	7992 adults	Fundus photography; CRAE	Arteriolar narrowing associated with increased future diabetes risk.
Rotterdam Study; Ikram 2006 [32]	2099 participants	Fundus photography; vessel diameters	Retinal arteriolar changes predicted impaired fasting glucose and diabetes.
Nguyen 2009 [34]	224 participants	Dynamic Vessel Analyzer; flicker response	Diabetes and DR associated with impaired flicker-induced retinal vasodilation.
Stefański 2023 [41]	76 T1D, 25 controls	SLDF; WLR, RCF	T1D associated with increased arteriolar WLR and wall thickness.
Ludovico 2003 [42]	47 T2D without DR, 20 controls	SLDF; capillary blood flow	Reduced retinal capillary flow detected before clinical DR.
Cristescu 2019 [36]	35 diabetes without DR, 21 controls	AO; WLR, wall thickness	Increased arteriolar wall thickness and WLR before visible DR.
Matuszewski 2022 [43]	51 T1D, 18 controls	AO; WLR, wall thickness	Retinal arteriolar remodeling correlated with diabetes duration.
Zaleska-Żmijewska 2017 [44]	12 prediabetes, 22 controls	AO; WLR, photoreceptors	Early neurovascular changes detectable before diabetes onset.
Hosein Nouri 2024 [45]	Review	OCTA; vessel density, FAZ, non-perfusion	OCTA detects early microvascular changes and DR biomarkers.
Huang & Fawzi 2024 [37]	Diabetes with preclinical and clinical DR	AO; arteriolar WT, WLR	Hypertension may drive early wall thickening; diabetes predominates in clinical DR.

## Data Availability

No new data were created or analysed in this study. Data sharing is not applicable to this article.
